# The Infirmities of Genius, Illustrated by the Habits and Constitutions of Men of Literature and Genius

**Published:** 1833-10-01

**Authors:** 


**THE INFIRMITIES OF GENIUS ILLUSTRATED BY REFERRING THE ANOMALIES IN THE LITERARY CHARACTER TO THE HABITS AND CONSTITUTIONAL PECULIARITIES OF MEN OF GENIUS. By *R. R. Madden*, Esq. Author of "Travels in Turkey," &c. 2 vols. 12mo. Saunders and Otley, 1833.**

PROVIDENCE has not left the good and the bad things of this world so much to chance, as some philosophers imagine. The balance of happiness and misery—of enjoyment and suffering—is adjusted by a hand unseen, and we very much doubt whether, all things considered, the scale ever turns very far from the equilibrium. We see and we envy the gifts of fortune and the possession of splendid talents; but the dark side of the picture is generally concealed from our view, and our estimates of prosperity and adversity are often most erroneous.

The Author of the work before us is well known to the public, and to our readers, by his travels in the East, of which we gave an ample account in this journal. As a medical man and a traveller, he has had opportunities of studying human nature on a wide scale, and in the present instance, he has turned his talents and acquirements into a channel where their force and utility will have every chance of success. The subject is far from being uninteresting to the medical practitioner. It is his duty and his interest, to study MAN in health, as well as in sickness—and to watch the workings of mind as well as of matter, in the human microcosm. Physicians (by which we mean medical men of all descriptions) have lost much in public estimation, by directing their attention too exclusively to disorders of the corporeal fabric, and by thinking it an extra-professional labour, to study the moral part of our nature. The physician, in fact, has infinitely greater, better, and more numerous opportunities of acquiring an intimate knowledge of metaphysics than the divine or the moral philosopher; and, whenever he has directed his attention to these subjects, he has excelled in them. The immortal Locke is a sufficient example.

With the last-mentioned physician and metaphysician, we disbelieve the doctrine of innate ideas, and conceive that all those peculiarities or excentricities of individuals, which are commonly attributed to mind, are in reality, connected with, or rather dependent on, certain peculiarities of bodily organization. But it will be said, "we see genius, talent, &c. hereditary in families." Well. This, to our minds, is a proof that talent and genius depend on organization. It would be a dangerous doctrine to maintain, that the *soul* is transmitted from parent to progeny! If a "*divinæ particula auræ*" be not conferred on every individual at the moment of his first creation, adieu to all hope of the immortality of the soul! When we observe genius or talent, then, transmitted hereditarily, we believe that it is the *capacity* in our corporeal organization for the future exercise of the soul, that is born with us. This doctrine, so far from tending to materialism, is, in fact, the grand bulwark against that doctrine. He who believes that the soul is transmitted from father to son, with peculiarities, like the colour of the hair or the form of the nose, is a downright materialist, and degrades the destinies of man.

But descending from this argument to a subject somewhat lower; we think it will hardly be disputed, that much of men's tempers, peculiarities, and even their disorders, depend on their avocations and pursuits. Among the lower classes—artizans, mechanics, &c. this fact is notorious; and there is no reason to think that science and literature are exempt from the same effects.

In respect to literary men, it is generally admitted, that they are an "irritable race," subject to many infirmities of mind and body, eccentricity being the "badge of all their tribe."

"The studious man sets out with stealing an hour or two from his ordinary repose; sometimes perhaps more; and finishes by devoting whole nights to his pursuits. But this night-work leads to exhaustion, and the universal sense of sinking in every organ that accompanies it, suggests the use of stimulants, most probably of wine; alcohol, however, in some shape or other. And what is the result?—why, the existence that is passed in a constant circle of excitement and exhaustion, is shortened, or rendered miserable by such alternations; and the victim becomes accessary to his own sufferings." 17.

These are extreme cases, but not exceedingly unusual. In a majority of instances, the man of literature or science consumes his strength and spirits in his study, without taking sufficient exercise in the open air—the consequence of which is, dyspepsia, with all its proteian horrors, and determination to the brain, the only organ in the body which is over-worked.

In the second chapter, Mr. Madden discusses the use and the abuse of literary habits and literary temperaments. The advantages of literature dissipating the evils of ennui, are humorously characterised in the following satirical sketch.

"It surely is not the least advantage of literary employment that it enables us to live in a state of blissful ignorance of our next-door neighbour's fortune, faith, and politics; that it produces a state of society which admits of no invasion on domestic privacy, and furnishes us with arms against *ennui*, which supersede the necessity of a standing army of elderly female moralists, and domestic politicians. In large cities, at least, literature occupies the ground which politics and scandal keep possession of in small ones; in the time of Tacitus the evil was common to the communities of both:

'Vitium parvis magnisque civitatibus commune
Ignorantium et invidiam.'

Leisure, it seems, had no better occupation ere 'the art of multiplying manuscripts through the intervention of machinery' was discovered; but in these days of book-publishing celebrity, when the Press pours volumes on the town with the velocity of Perkins' steam-gun, one has hardly sufficient leisure to acquire a knowledge even of the names of those 'dread counterfeits' of dead men's thoughts, which living plagiarism is continually recasting and sending forth." 24.

But we must pass over several chapters, in order to notice the sixth—The Influence of Studious Habits on the Duration of Life. It is remarked by D'Israeli that, "every class of genius has distinct habits—all poets resemble one another, as all painters, and all mathematicians. There is a conformity in the casts of their minds, and the quality of each is distinct from the other."

"For the purpose," says Mr. M., "of ascertaining the influence of different studies on the longevity of authors, the tables which follow have been constructed, in which the names and ages of the most celebrated authors in the various departments of literature and science are set down, each list containing twenty names of those individuals who have devoted their lives to a particular pursuit, and excelled in it. No other attention has been given to the selection than that which eminence suggested, without any regard to the ages of those who presented themselves to notice." 71.

A little reflexion will convince us that these tables are liable to great error; for we never can get a true estimate of longevity, without having the whole number in a class, instead of a few of the most eminent. Thus, suppose we were to take 20 of the most distinguished English physicians or surgeons, on the rolls of fame, and divide the aggregate of their ages by 20. It would certainly give us the average longevity of our most distinguished men in the two departments, but it would present no criterion for the duration of life, in physicians and surgeons generally—though hundreds of them might have been as studious and skilful as any of those selected in the list of twenty, but who were not fortunate enough to have their names transmitted to posterity in print. Still, the tables here collected by Mr. Madden are curious, and the resumée we shall present to our readers.

| | *Aggregate years.* | *Average years.* |
|---|---|---|
| Natural Philosophers | 1504 | 75 |
| Moral Philosophers | 1417 | 70 |
| Sculptors and Painters | 1412 | 70 |
| Authors on Law and Jurisprudence | 1394 | 69 |
| Medical Authors | 1368 | 68 |
| Authors on Revealed Religion | 1350 | 67 |
| Philologists | 1323 | 66 |
| Musical Composers | 1284 | 64 |
| Novelists and Miscellaneous Authors | 1257 | 62½ |
| Dramatists | 1249 | 62 |
| Authors on Natural Religion | 1245 | 62 |
| Poets | 1144 | 57 |

From these tables it would appear, that those pursuits in which the imagination is strongly exerted, are unfavourable to longevity. The natural philosophers, for instance, out-lived the poets by 18 years on an average! Is natural philosophy a less laborious study than poetry? Or is it that the latter is rather a passion than a pursuit, communicating an excitement to all our feelings and faculties that wears down the vital powers much sooner than the most profound reflection on subjects of a different character?

Mr. Madden has several chapters on the different classes above-mentioned, which will richly repay perusal. The 13th chapter is on the last moments of men of genius.

"Generally speaking, the influence of literature and science over the mind and the demeanor of men, is at no period displayed to such advantage as at that of the close of life. What medical man has attended at the death-bed of the scholar, or the studious man, and has not found death divested of half its terrors by the dignified composure of the sufferer, and his state one of peace and serenity, compared with the abject condition of the unenlightened mind in the same extremity? Those, perhaps, who relinquish life with the most reluctance, paradoxical as it may appear to be, are to be found in the most opposite grades of society—those in the very highest and lowest walks of life." 138.

The fact is, that it is in health, and in diseases not necessarily fatal, that people are most apprehensive of death. When the last scene arrives very few are aware of their danger. Their friends and themselves entertain hope till insensibility takes place, and then the departing spirit knows nothing of the event that is impending.

"In different countries, likewise, it is singular in what different degrees people are influenced by the fear of eternity, and in what different ways the pomp of death, the peculiar mode of sepulture, reasonable views of religion, and terrifying superstitions affect the people of particular countries. The Irish, who are certainly not deficient in physical courage, support bodily suffering, and encounter death, with less fortitude than the people of this country. A German entertains his fate, in his dying moments, more like a philosopher than a Frenchman. And, of all places in the world, the capital of Turkey is it, where we have seen death present the greatest terrors, and where life has been most unwillingly resigned. The Arabs, on the other hand, professing the same religion as the Turks, differ from them wholly in this respect, and meet death with greater indifference than the humbler classes of any other country, Mahomedan or Christian. It is truly surprising with what apathy an Arab, in extremity, will lay him down to die, and with what pertinacity the Turk will cling to life—with what abject importunity he will solicit the physician to save and preserve him." 140.

In the expedition of Ibrahim Pasha to the Morea, when Mr. Madden accompanied him, and where he had opportunities of seeing hundreds dying daily in the camps, the haughty Moslem went to the society of his celestial Houries, like a miserable slave, while the good humoured Arab went like a hero to his long last home.

Mr. Madden, towards the middle of the first volume, illustrates his observations on the character of poets, by examining minutely into the temperaments and other physical conditions of some of the more eminent individuals of that race.

Pope stands the first on the list; and our author very successfully explains the peevishness of temper and way-wardness of humour, evinced by the immortal Bard of Twickenham, by his bodily infirmities. Johnson remarks that, "by natural deformity, or accidental distortion, the vital functions of Pope were so much disordered, that his life was a long disease." The deformity resulted from an affection of the spine, contracted in infancy, and, no doubt, occasioning the extreme delicacy of his constitution. The disorder of his digestive organs, too, had a great and preponderating influence on his temper, as every practical physician knows. Pope is represented by his biographer, as irascible, capricious, peevish, and resentful —all which manifestations of the temper spring from corporeal disorder, and especially disorder of the digestive organs.

"Johnson has pictured Pope as he really appeared to the world; but Bolingbroke spoke of him when he was on his death-bed, not as he appeared to be, but as he knew him to have been, when he said to his weeping attendants,—'I have known him these thirty years; he was the kindest-hearted man in the world.' Who knows under what paroxysm of mental irritation of that disease which, more than any other, domineers over the feelings of the sufferer, he might have written those bitter sarcasms which he levelled against his literary opponents? Who knows in what moment of bodily pain his irascibility might have taken the form of unjustifiable satire, or his morbid sensibility assumed the sickly shape of petulance and peevishness? Who knows how the strength of the strong mind might have been cast down by his sufferings, when 'he descended to the artifice' of imposing on a bookseller, and of 'writing those letters for effect which he published by subterfuge?' Who, that has observed how the vacillating conduct of the dyspeptic invalid imitates the vagaries of this proteiform malady, can wonder at his capriciousness, or be surprised at the anomaly of bitterness on the tongue, and benevolence in the heart, of the same individual." 186.

Allusion is made to Byron, who spared neither friends nor foes when his angry mood was on him. Mr. M. justly observes, that there was far more malignity in the publication of those lines on Rogers, than in the original penning of them. They were probably written under the influence of bodily disorder, but they were published in cold and calculating blood.

The character of SAMUEL JOHNSON is admirably descanted on in a physiological and pathological point of view. We can only make room for one short extract.

"Johnson's disorder (if we may be allowed the expression) had three phases, the character of each of which distinguished a particular period of his career, or rather predominated at a particular period, for it cannot be said that the hues of each were not occasionally blended. At twenty, however, his despondency was of a religious kind: about forty-five 'his melancholy was at its meridian,' and then had the shape of a fierce irritability, venting itself in irascibility of temper, and fits of capricious arrogance.

At the full period of 'three-score years and ten,' the leading symptom of his hypochondria was, 'the apprehension of death, and every day appeared to aggravate his terrors of the grave.' This was 'the black dog' that worried him to the last moment. Metastasio, we are told, never permitted the word death to be pronounced in his presence; and Johnson was so agitated by having the subject spoken of in his hearing, that on one occasion he insulted Boswell for introducing the topic; and in the words of the latter, he had put 'his head into the lion's mouth a great many times with comparative safety, but at last had it bitten off.'" 231.

For many years before his death the prospect of dissolution was most terrible to this great-minded man. He acknowledged to Boswell that he never had a moment in which the idea of death was not terrible to him. In the intervals of conversation he was often heard to repeat the following line from Shakespeare—

"To die, and go we know not where."

It is not a little curious, that, to the sceptical mind, death is nearly as dreadful as to the superstitious. The idea of annihilation is little less abhorrent to human nature than that of damnation. Johnson shewed the ruling passion, or rather fear, till the final moment. On the last day of his existence, he declared that he would give one of his legs for another year's life! We do not quite agree with our author in the following sentiment.

"If Johnson's fear of death were not the effect of disease, it would be impossible to contemplate his conduct either in sickness or in sorrow, in his closet or on his death-bed, without feelings of absolute disgust. What other sentiment could be entertained—

"For him who crawls enamoured of decay,
Clings to his couch, and sickens years away." 235.

People of the strongest intellect, and of the most religious and moral character, may entertain ideas respecting death and another state of existence, that may embitter life, and yet without any disease to account for the phenomenon. Education has great influence on our feelings respecting dissolution—nay, even the history of horrid tales, as of resuscitations in the grave, have, to our knowledge, rendered many people wretched, whose healths were not affected. We have no doubt that Johnson laboured under hypochondriasis, and that his abhorrence of death was heightened by this unhappy condition of the constitution; but we believe that the above-mentioned dread was connected with some moral feeling, tenet, or superstition, quite independent of corporeal malady. His belief in ghosts cannot be fairly attributed to hypochondriasis, when we find it as firmly implanted in the minds of the rudest shepherds of the Grampian mountains, where hypochondria never shewed its face. We are more inclined to agree with Boswell, that many of Johnson's superstitions and weaknesses were the weaknesses of a pious and good man, and were "the result of early religious impressions instilled into his mind by his mother, with assiduity, not with judgment." Sunday, he said, was a heavy day for him. When a boy, he was confined on that day, to the perusal of "The Whole Duty of Man," from a great part of which he could derive no instruction.

Mr. Madden appears to think, that Johnson's death was hastened by injudicious bleeding for a spasmodic asthma. It has escaped him that, in a former number of this Journal, we alluded to the *post-mortem* examination of the great lexicographer, which, we believe, was never published, but which we know through the gentleman who opened his body—a gentleman now living in London. His lungs exhibited a very singular appearance—some of the air-cells being as large as a hen's egg, and the whole of the lungs being studded with uncountable numbers of these dilatations, varying in size from a hen's egg to a mustard seed. We lately met with a similar instance—and it was during the examination of the body that Mr. Thomas, of Leicester Place, related the above-mentioned circumstance of Johnson, whose body he had dissected.

Burns, whose works were once fashionable among all ranks of society, has lately declined in popularity among the aristocracy. Lord Byron, before he took to drink so largely of gin, could not bear to think of the ungentlemanly habits of poor Burns, who solaced his toils and disappointments with a glass of whiskey and a pipe of tobacco. In the eyes of the proud Lord, therefore, the Bard of the North was "a strange compound of dirt and Deity." In fashionable morality it is one thing to get drunk with claret or champagne, and quite another to addle the brain with porter or brandy. The one is merely a little excess in the "pleasures of the table" —the other is "disgusting intoxication." There is no doubt that winebibbing is less injurious and more gentlemanly than dram-drinking; but in this country the poor man is prohibited from wine, on account of its price, otherwise he would, in all probability, prefer wine, if it were as plenty and as cheap as in France. In Burns' time, also, intemperance was more common and less reprehended than in ours. Cowley died by lying out drunk in a field one night. Dryden drank hard, in after-life; but wine had no exhilarating effect—while with Byron, it made him "savage instead of mirthful." Parnel, according to Pope, "was a great follower of drams, and strangely open and scandalous in his debaucheries." Churchill was found drunk on a dunghill—Prior was very intemperate, and Pope himself, (according to Dr. King) "hastened his end by drinking spirits." It is true that these illustrious precedents among the Muses, are no excuse for Burns' irregularity; but it is to be remembered that Burns was temperate while at the plough, and only got accustomed to excesses when he became an author and exciseman. He was affected with intense dyspepsia long before he began to drink inordinately, and probably it was the wretched feeling of hypochondriasis which drove him to the bottle.

"The rapid progress of his disorder, both bodily and mental, is exhibited in the desponding tenor of his letters, from the period of his relinquishing his agricultural pursuits. Indolence, the baneful attendant of morbid sensibility, aggravated his hypochondria. Idleness became preferable to a distasteful occupation; and idlenesss, as usual, was followed by miseries which rendered existence intolerable without excitement." 287.

His amiable biographer, the late Dr. Currie, has traced the physical and moral deterioration of the Scotch Bard, with an able pen. In hypochondriasis extremes meet. The most ludicrous lines that Cowper ever wrote were indited under the most dolorous feelings. Such bursts of vivacity are by no means incompatible with the deepest gloom. The following picture of himself is by no means misplaced in a medical journal.

"I have been for some time pining under secret wretchedness; the pang of disappointment, the sting of pride, and some wandering stabs of remorse settle on my vitals like vultures, when my attention is not called away by the claims of society, or the vagaries of the muse. Even in the hour of social mirth my gaiety is the madness of an intoxicated criminal under the hands of the executioner." 291.

In another place, writing to Mr. Cunningham, he speaks of "his constitution being blasted, *ab origine*, with a deep incurable taint of melancholy that poisoned his existence." Dr. Currie remarks that—

"Burns, though by nature of an athletic form, had in his constitution the peculiarities and the delicacies that belong to the temperament of genius. He was liable, from a very early period of life, to that interruption in the process of digestion which arises from deep and anxious thought, and which is sometimes the effect, sometimes the cause, of depression of spirits. Connected with this disorder of the stomach, there was a disposition to headache affecting more especially the temples and eye-balls, and frequently accompanied by violent and irregular movements of the heart. Endowed by nature with great sensibility of nerves, Burns was in corporeal, as well as in his mental system, liable to inordinate impressions—to fever of body as well as of mind. This predisposition to disease which strict temperance and diet, regular exercise and sound sleep, might have subdued, habits of a very different nature strengthened and inflamed." 294.

The foregoing observations of Dr. Currie, are deserving the attention of all literary as well as medical men. They may there learn that excess in wine is not the only species of intemperance—and that excessive application to study is no less injurious than inordinate potations of drink.

In October, 1795, Burns, while returning from a tavern, benumbed and intoxicated, caught cold, ending in acute rheumatism, from which he partially recovered; but in the course of the next Summer, though still labouring under the chronic form of the disease, and, most probably, under affection of the heart, at that time not well understood, he was advised by his physicians to go to the sea and bathe there! He followed the advice—and was soon a corpse!

"Thus perished Burns in his thirty-seventh year. Let those who are without follies cast the first stone at his infirmities, and thank their God they are not like the other poor children of genius, frail in health, feeble in resolution, in small matters improvident, and unfortunate in most things." 298.

We have now concluded the first volume of this entertaining and instructive work. The second volume is wholly occupied with a medico-physical examination of the characters or rather temperaments of three distinguished authors—Cowper, Byron, and Scott.

The life of Cowper is more than usually interesting to the medical practitioner, since his character has been tried by all the tests that morality can apply to it, while the corporeal malady which occasioned or influenced his hallucinations, is left unnoticed by his biographers—most of whom were clergymen, and the rest laymen, who were incapable of elucidating the mystery of the Poet's religious depondency, which they have, therefore, left in the same obscurity in which they found it.

This unfortunate being appears to have been of a sickly and infirm constitution from his infancy. At a public school he was tyrannized over by a rascally miscreant, who, being stronger in body than Cowper, kept him in perpetual terror by his conduct. This is the bane of public schools. He was next put, very injudiciously, to the study of the law, and it was in the Temple that he experienced the first attack of a disorder that harassed him through life. This was, as he expressed it, "such a dejection of spirits as none but those who have felt the same can have the least conception of. Day and night I was upon the rack, lying down in horror, and rising up in despair." Who, that knows any thing of dyspepsia, will not acknowledge that this was just as much a corporeal malady as a fever or a toothache? The classics had now no charm for him—change of scene was recommended—and at Southampton the first symptom of monomania became developed—and that on the worst of all subjects, as far as the hope of cure is concerned. On a clear and beautiful morning, the scenery and the refreshing atmosphere inspired a kind of paroxysm of internal pleasure, such as a poetical imagination very often feels, under similar circumstances. He attributes this delight to the Almighty fiat—and to nothing less. But, unfortunately, this transitory emotion of religious joy was only the precursor of future remorse and misery. In fact, the mind was unpoised by corporeal disorder, before this out-break, which was only the first obvious or unequivocal demonstration of the melancholy truth.

"Satan," he says, "and his own wicked heart, quickly persuaded him that he was indebted for his deliverance to nothing but a change of scene, and the amusing varieties of the place; and by this means had turned the blessing into a poison." 20.

From this time his mind became distracted with religious doubts, and ultimately with remorse. He believed that he had committed the "unpardonable sin," and that he had incurred the dreadful penalty of eternal reprobation, for neglecting to improve to his advantage the communion of his sinful spirit with the Almighty at Southampton!!

Alas! how many thousands, or rather millions of our fellow-creatures are torturing their imaginations with similar hallucinations respecting the divine and benevolent author of our existence, who sent us into this world with senses to enjoy the gifts of nature profusely scattered around us, and with a mild religion that prohibits no innocent enjoyment of them! In a religious point of view, they are as insane as Cowper; and they only want (too many of them not long) the corporeal infirmities of the Bard to render them fit subjects for the lunatic asylum!

In every future paroxysm of his disorder, the terrific notion that, by his misconduct on this occasion, he had forfeited every claim to salvation, became the undeviating theme of his madness. It was his misfortune to be always surrounded by what are called *serious* characters—many of them such pests of society, that they embitter life in this world, without giving the soul any better chance of happiness in the next! We make not this reflection on them in the way of *condemnation*, but of pity for themselves and their disciples. We have no doubt of the honesty and sincerity of their intentions and beliefs; but we consider them as all insane, *in degree*, though, in this world, they are not likely to be convinced of their error, or persuaded to relinquish it.

A situation as Clerk in the House of Lords having been procured for him, he was terrified at the idea of appearing in public, and threw up the place. Though frightened at the thoughts of death hitherto, he now longed for it, and entertained an intention of suicide! He was now decidedly insane; but no medical advice was procured. A divine came, and urged the monomaniac to a "lively faith,"—to which poor Cowper answered—"most earnestly do I wish it would please God to bestow it on me"! As may easily be supposed, these religious conversations and interviews only aggravated the disorder. At length he was placed in a private lunatic asylum, under the care of Dr. Cottin, 1763. In two years he was so much improved as to be left at large, and his mind restored to comparative tranquillity. But, unfortunately, he now placed himself in the family of the Rev. Mr. Unwin, where he was debarred from all society, except that of the SERIOUS! This step had baneful consequences. In 1773, he had another outrageous *paroxysm* of monomania (for we consider that he was never entirely free from it) similar to that at Southampton. He continued to suffer unmitigated miseries for five years, after which his reason was at length restored. From this time till he attained the age of 50 years, his malady assumed the character of mild melancholy, with occasional paroxysms of a graver nature. At the age of 50, he became an author—no great proof, it will be said, of a sound mind! Contrary to expectation, he appeared before the public in this new and arduous character, with little or no anxiety. In 1782, he fortunately became acquainted with Lady Austin; and in her society he found great delight. His dejection became quickly banished, and his health evidently improved. Had such an event happened 20 years sooner, the consequences might have been most happy! She asked him to write a poem in blank verse. "What is to be the subject?" said he. You can write upon any thing, was the answer—write on the sofa where you now sit. The result is well known. Had he had such a friend at an earlier period, the history of his life would have assumed a different complexion. But he parted from Lady Austin, and Mrs. Unwins died. So did his father and brother; but with few symptoms of sorrow on the part of the hypochondriac, whose own feelings of misery were too intense to permit much participation in the fate of others.

Among the misfortunes of Cowper was that of living nearly 20 years at

Olney, in an atmosphere tainted with malaria! In 1787, he had another acute paroxysm of monomania, evidently connected with corporeal disorder —especially affection of the head, accompanied, or rather preceded, by giddiness and pain. The following passage from Mr. Madden is deserving of quotation.

"It is well worthy of observation, that in this and every other similar attack of his dreadful depression, head-ache and giddiness are spoken of as the premonitory symptoms of his disorder. But it does not appear that local depletion, or any other effective means, were ever resorted to, to obviate or prevent his sufferings, which were evidently the effects of determination of blood to the head, or probably the chronic effects of that determination—of effusion and pressure on the brain—the not unlikely source of all his miserable feelings. On one of these occasional attacks, the composition of theological essays are recommended to him; on another, the translation of spiritual songs; on another, the production of a volume of original hymns; but at any of these periods the services of a cupper, and the judicious care of a physician, might have proved of more advantage." 83.

He had scarcely recovered from this attack, when the Rev. Mr. Bull, (one of his *serious* friends, or rather *destructive* enemies) importuned the religious monomaniac to compose a set of hymns!! "Ask possibilities (replied Cowper) and they shall be performed; but ask not hymns from a man suffering with despair, as I do. I would not sing the Lord's song, were it to save my life." We are tempted to go farther into the life of Cowper than we otherwise should, because there are, in our own profession, an increasing number of "psalm-singers," who would zealously pursue the same course of conduct which destroyed Cowper! Muddle-pated, narrow-minded, bigotted, enthusiastic—perhaps hypocritical physicians, who are, for ever, thrusting their religious dogmas into the minds of their patients, while drenching their bodies with physic! We have no patience with such personages. Those who are sincere, are fools—those who are not so, are rogues.

In the beginning of 1791, he had another attack. His translation of Homer had mitigated his sufferings, and the publication brought him into acquaintance with Hayley, who avers that Cowper, now in his 61st year, appeared to feel none of the infirmities of advanced life—being active and vigorous in mind and body. In January, 1794, his forebodings were realized. A severer attack than any that preceded, paralyzed his mental powers, and they never afterwards recovered. He lingered out a miserable existence, and died of dropsy, in May 1800, aged 69 years.

The conclusion is easily summed up. From his earliest infancy, Cowper was of a delicate constitution and timid disposition. Excessive application to professional studies in the Temple, injured his health, and disturbed the functions of the nervous system, so as to lay the foundation for that morbid sensibility which rendered his life a scene of wretchedness. Hypochondriasis ensued, and mounted into occasional paroxysms of acute monomania. In the intervals, he was rarely, if ever, free from chronic monomania of the religious kind. Finally, he was most unfortunate in having those around him, who aggravated his mental malady by the most injudicious arguments and reasonings. Had he been a poor man, and confined in a lunatic asylum, he would soon have been cured. Or, as it was, had he lived in the present day, he would have got well under proper medical treatment, and by protection from the society of *serious* persons!

### BYRON.

It is not our intention to dip far into the *infirmities* of this genius. Byron soared too high for our optics to follow his ætherial flights about the summits of Olympus. If Cowper was religiously mad, Byron was irreligiously so:—if the former was a fanatic, the latter was a sceptic, if not something worse. Cowper was always repenting of sins that he had never committed —Byron had probably little regret, except at the inability of indulging in what Cowper would have considered unpardonable sins.

We have no doubt that Byron's eccentricities, and his real or feigned misanthropy depended much on some corporeal disorder, though that elucidation seems never to have entered the minds of his biographers.

"His symptoms have indeed been noticed under various names, when productive of any extraordinary and palpable effect, but they have been so indefinitely described, that nothing but medical investigation is competent to a solution of the difficulties they present. In one place we read of his being subject to an hysterical affection, in another of his being carried out of a theatre in a convulsive swoon; elsewhere, of an apoplectic tendency, attended with temporary deprivation of sense and motion; at another time, of nervous twitches of the features and the limbs, following any emotion of anger, and from trivial excitement, and slight indisposition, of temporary aberrations of intellect, and delirium; but no where do we find the cause of these phenomena plainly and intelligibly pointed out, nor the real name given to his disorder, till his last and fatal attack. The simple fact is, he laboured under an epileptic diathesis, and on several occasions of mental emotion, even in his early years, he had slight attacks of this disease. If feelings of delicacy induced his biographers to conceal a truth they were aware of, or deemed it better to withhold, their motive was unquestionably a good one; but it was nevertheless a mistaken delicacy; for there are no infirmities so humiliating to humanity as those irregularities of conduct in eminent individuals; and the only palliation they admit of is often precluded by our ignorance of the bodily disorders under which they may have laboured." 130.

This epileptic diathesis was probably hereditary, and derived from his ill-tempered mother. He had not regular recurrences of the complaint; which, like masked gout, only produced slight seizures, and a host of anomalous phenomena, when the mind was excited, or the body weakened by excesses. "In boy-hood, the most trivial accident was capable of producing sudden deprivation of sense and motion." Two months before his death, he had a regular and severe convulsion, in the presence of Captain Parry, and in Colonel Stanhope's room. With such a morbid diathesis, a devotion to literature, and especially to poetry, would be very apt to produce that melancholy, morbid sensibility, and sarcastic temper, so conspicuous in Byron. For such a man society had few charms. He made a merit of his aversion from social intercourse, and prided himself on being independent of the frivolous amusements of the world. His self-concentration led him to think his own mind all-sufficient for his individual felicity—and a refined selfishness became the most prominent feature of his isolated feelings.

"The question of Byron's hypochondria no one can dispute, who has perused his journals. Its various Protean forms are there set forth in language which affectation could not forge, nor fiction mimic. 'What can be the reason,' he says in his journal, 'I awake every morning in actual despair and despondency? In England, five years ago, I had the same kind of hypochondria, but accompanied with so violent a thirst, that I have drank as many as fifteen bottles of soda-water in a night, after going to bed.' This unaccountable dejection without a cause, this constant waking in low spirits, he frequently alludes to, and expresses an apprehension of insanity; in his own words, of 'dying like Swift, at the top first.'" 157.

There is as little doubt that Byron's disorder was greatly aggravated by errors of diet, and inattention to proper regimen. On this last subject, indeed, he entertained the most ridiculous notions. He believed the rigid abstemiousness of an anchorite to be compatible with the most profuse expenditure of nervous energy—and that the exhaustion of the mind was only to be balanced by exertion of the body. He sallied, however, from one extreme to the other—fasting whole days, or living on bread and cheese—and then drinking "five fathoms deep," and plunging into excesses of various kinds, without limit. Latterly, indeed, when harassed by an irregular intermittent or remittent fever, Lord Byron appears to have acted with more than usual imprudence. He took a cold sea-bath in January, and in the night, and continued in the water for a considerable time. After this he was soon seized with rheumatic fever. It appears, too, that he was in the habit of taking opium, by which the tone of his stomach was injured. He was, in fact, a martyr to indigestion. In his last illness, to slow and irregular remittent—perhaps rheumatic fever, was added, if the history and dissection be fairly detailed, inflammation of the brain. We believe he was injudiciously treated—but we do not agree with Mr. Madden, that his death was occasioned by abstinence and bleeding. Few, indeed die of such a cause; but thousands fall victims, annually, to want of depletion and too much sustenance. In Lord Byron's case, the depletion was too late—and consequently was useless, perhaps injurious. The medical treatment, in other respects, was vacillating and inert. He had no confidence in his medical attendants, and they, of course, had no influence or control over his conduct. Be this as it may, the life of the celebrated Bard was embittered by corporeal ailments, and there is no doubt that his temper was soured by the same. The tenor of his writings was tinctured by the state of his health; and had the poet been free from corporeal and mental irritation, the probability is, that his writings would have assumed an entirely different complexion, breathing sentiments of philanthropy and piety, instead of misanthropy and infidelity.

### Sir Walter Scott.

When we came to the last character introduced by Mr. Madden as an illustration of the "infirmities of genius," we closed the book and began to ponder in our own minds. We asked ourselves, "what are the incidents in the life of the Northern Wizzard, that can furnish food for Mr. Madden on this point?" We could not immediately call to mind any proofs of *infirmity* in the Caledonian Bard, except two—the committal of his property to the care of a bookseller, and his health to the influence of a Sirocco or Tramontane at Naples. On these two points we have no doubt that Sir Walter was insane—or, at all events, that he evinced "infirmity of genius," to a most deplorable extent. This was the more wonderful in a Scotchman, whose head always displays the organ of *prudence*, or, as the Phrenologists will have it, the organ of *acquisitiveness*, in a state of extreme development. . . . . . . . . . .

On recurring to Mr. Madden, we found that he winded up his illustrations of the "infirmities of genius," by adducing an instance where these infirmities were almost entirely absent. The life of Sir Walter Scott is brought forward as an example for literary men to imitate—as a specimen where the labours of the mind are combined with relaxation or salutary exercise of the body, so as to counteract the injurious effects of study. But there were circumstances in the physical as well as the moral constitution of Scott, which enabled him to go through immense literary labour without injury to his health.

"Scott's sensibility," says Mr. M. "fortunately for his felicity, was not of that intense description that its tranquillity was staked on the hazard of his literary success, or that the labour of composition was coupled with the anxieties of authorship, the ardour of enthusiasm, or the ecstasies of successful genius. In this respect Scott had the decided advantage over the majority of the *genus irritabile* of authors, whether of works of prose or poetry. Pope could not proceed with certain passages of his translation of Homer without shedding tears. Metastasio was found weeping over his Olympiad. Alfieri speaks of a whole act in one of his plays written under a paroxysm of enthusiasm, weeping while he wrote it. Dryden was seized with violent tremors after the composition of his celebrated ode. Rousseau, in conceiving the first idea of his Essay on the Arts, felt the disturbance of his nervous system approaching to delirium. Buffon could not enter on a work which absorbed his faculties, without feeling his head burn, and his features becoming flushed. Beattie, after the completion of a volume of metaphysics, never had the courage to look into the book when it was printed, so great was the horror of his undertaking. Goldoni says he never recovered from the exhaustion of his spirits after the production of sixteen comedies in one year. Smollet by over-excitement disordered his brain, and laboured for six months under a coma vigil. These and many other instances have been enumerated by D'Israeli in his admirable work. Scott, however, was luckily exempt from the excitement of such morbid feelings, and from the delusions which are the consequences of them. It is but a step, it is said, which separates the fervour of enthusiasm from the frenzy of insanity, and not unfrequently are the children of genius found tottering on the verge of that calamity. Tasso held a conversation with a spirit gliding on a sun-beam, and we are told by Thuanus, he was frequently seized with fits of distraction which did not prevent him writing excellent verses. Malebranche heard the voice of God distinctly within him. Lord Herbert interrogated the Deity about the publication of his book, and in a kneeling posture calmly awaited the reply. Pascal often started from his chair at the appearance of a fiery gulf opening by his side. Luther conversed with demons, and on one occasion threw an inkstand at the devil's head, an action which his German commentator greatly applauds, because there is nothing the devil hates so much as ink. Descartes, after long seclusion, was followed by an invisible person calling on him to pursue the search of truth. Swedenburgh not only walked over Paradise, but has given a description of the fashion of the houses; but the glorious egotism of Benvenuto Cellini, says D'Israeli, outstripped the visions of all his predecessors, for he was accustomed to behold a resplendent light hovering over his own shadow." 221.

From all these illusions Sir Walter was free. But the mania of speculation—the mental epidemic of 1825—reached even to Abbotsford. The rage for building, embellishing, planting, and collecting objects of antiquity, led to an expenditure of fifty-thousand pounds—and the embarrassment compelled the Bard of the North to have recourse to other than literary means of increasing his income. Fortune, in short, began to be weary of her smiles, and the long unclouded horizon of Sir Walter became darkened by adversity. The noble resolution of working for his creditors, after this period, enhanced his moral character, but diminished his fame, and ultimately put a period to his existence. He was thenceforth a literary slave—toiling with his intellect to repair the losses occasioned by the imprudence of others! It was a state of existence far worse than that of the African in the West Indies. When *his* daily labour is over, he has no care—no thought—no anxiety or sorrow. But to work by the midnight lamp, exhausting our intellectual energies for the profit of others, is a terrible species of slavery, and the effects were soon visible in the literary labours of Sir Walter Scott! Every successive publication was a fainter emanation of his extraordinary genius, and the last of his productions was the feeblest gleam of its departing glory!

To recruit his health he was kindly, but imprudently urged to make a tour in Italy. With an organic disease of the brain (ramollissement) nothing could be more injudicious than travelling through a land where the elements of moral and physical excitement are so numerous and concentrated. The consequences were such as might have been expected. His health was improved by the voyage to Naples; but his journey through Italy and homeward completed the tragedy. A second paralytic seizure, while descending the Rhine, put the finishing stroke to the malady in the brain, and from it he never recovered.

We have done as much justice to Mr. Madden as could reasonably be expected in a Medical Review. The work is a desideratum for the literary classes of society, and we strongly recommend it to the patronage of our professional brethren, as well entitled to their attention.

